# Impact of psychiatric comorbidities on emergency surgical patients’ outcomes

**DOI:** 10.1192/bjo.2021.647

**Published:** 2021-06-18

**Authors:** Hongying Chen, Michael Devine, Waqar Khan, Iqbal Z Khan, Ronan P Waldron, Michael Kevin Barry

**Affiliations:** Department of Surgery, Mayo University Hospital, Ireland

## Abstract

**Aims:**

Psychiatric disorders are increasingly prevalent and present as a comorbidity in many hospitalized patients. Studies have demonstrated that the presence of comorbid psychiatric conditions (CPC) is associated with worsened inpatient outcomes. Emergency surgical admissions and the impact of CPC on their outcomes has not been studied in Ireland to date. This study aims to provide a comprehensive analyses of the relationship between a wide range of psychiatric comorbidities and surgical presentations.

**Method:**

The Hospital In-Patient Enquiry (HIPE) and prospectively maintained electronic patient records were used to identify all surgical emergency admissions between 31st August 2019 and 1st September 2020 to Mayo University Hospital, Ireland. Patient demographics, comorbidities, primary diagnoses, length of stay (LoS), discharge destination, and surgical interventions were recorded. Subgroup analyses were performed examining LoS variation in the type of surgical presentation. Physical comorbidities were scored using the Charlson Comorbidity Index (CCI). Statistical calculations were performed using SPSS.

**Result:**

A total of 995 admissions were recorded. The presence of CPC increased the overall mean LoS by 1.9 days (p = .002). This trend was observed in both operative and conservative management. Significant increase in LoS was noted in patients with a comorbid depression (2.4 days, p = .003), dementia (2.8 days, p = .019), and intellectual disability (6.7 days, p = .007). Subgroup analysis revealed greater LoS in patients with CPC diagnosed with non-specific abdominal pain (1.4 days, p = .019), skin and soft tissue infections (2.5 days, p = .040), bowel obstruction (4.3 days, p = .047), and medical disorders (18.6 days, p = .010). The odds of nursing home or convalescence as a discharge destination was 2.44 (95% CI: 1.37–4.35, p = 0.002) in patients with CPC and the odds of self-discharge against medical advice in this population was 4.89 (95% CI: 1.43–16.70, p = 0.005). No significant difference was observed in mortality and readmission rates.

**Conclusion:**

Psychiatric comorbidities significantly impact length of hospital stay and influence discharge planning in surgical inpatients. Greater vigilance is required in providing care for patients with psychiatric comorbidities, particularly those with depression, dementia and intellectual disability. Better optimisation of facilities and a more personalised approach to patients with CPC are required to improve inpatient outcomes and resource allocation.

